# Fatigue in Type 2 Diabetes: Impact on Quality of Life and Predictors

**DOI:** 10.1371/journal.pone.0165652

**Published:** 2016-11-08

**Authors:** Rupali Singh, Cynthia Teel, Carla Sabus, Patricia McGinnis, Patricia Kluding

**Affiliations:** 1 Department of Physical Therapy, The Sage Colleges, Troy, New York, United States of America; 2 School of Nursing, University of Kansas Medical Center, Kansas City, Kansas, United States of America; 3 Department of Physical Therapy and Rehabilitation Science, University of Kansas Medical Center, Kansas City, Kansas, United States of America; 4 Department of Physical Therapy, Stockton University, Galloway, New Jersey, United States of America; 5 Department of Physical Therapy and Rehabilitation Science, University of Kansas Medical Center, Kansas City, Kansas, United States of America; Charite Universitatsmedizin Berlin, GERMANY

## Abstract

Fatigue is a persistent symptom, impacting quality of life (QoL) and functional status in people with type 2 diabetes, yet the symptom of fatigue has not been fully explored. The purpose of this study was to explore the relationship between fatigue, QoL functional status and to investigate the predictors of fatigue. These possible predictors included body mass index (BMI), Hemoglobin A1C (HbA1C), sleep quality, pain, number of complications from diabetes, years since diagnosis and depression. Forty-eight individuals with type 2 diabetes (22 females, 26 males; 59.66±7.24 years of age; 10.45 ±7.38 years since diagnosis) participated in the study. Fatigue was assessed by using Multidimensional Fatigue Inventory (MFI-20). Other outcomes included: QoL (Audit of Diabetes Dependent QoL), and functional status (6 minute walk test), BMI, HbA1c, sleep (Pittsburg sleep quality index, PSQI), pain (Visual Analog Scale), number of complications, years since diagnosis, and depression (Beck’s depression Inventory-2). The Pearson correlation analysis followed by multivariable linear regression model was used. Fatigue was negatively related to quality of life and functional status. Multivariable linear regression analysis revealed sleep, pain and BMI as the independent predictors of fatigue signaling the presence of physiological (sleep, pain, BMI) phenomenon that could undermine health outcomes.

## Introduction

Fatigue is a word used very frequently in everyday conversations that has varied subjective meanings. Words like fatigue, tiredness, lacking energy and exhaustion are commonly used interchangeably. Definitions of fatigue also vary greatly in literature because defining fatigue is a challenge due to poor differentiation between causes, indicators and effects [1]. For this study, we defined fatigue as a multicausal, multidimensional sensation that includes physiological, psychological, and situational components [2–7].

Fatigue is a persistent and distressing complaint among people with type 2 diabetes [8, 9]. While fatigue also occurs in other medical disorders, the importance of fatigue may be greater in individuals with type 2 diabetes due to the complex management strategies that must be continually maintained, impacting the quality of life (QoL) of these individuals. QoL may also be negatively affected by hyper/hypoglycemia [10], number of complications [11–14], and depression [15]. Decline in functional capacity due to fatigue has been seen in patients with other chronic disorders like cancer and Parkinson’s disease [16–18]. It appears that fatigue may have a similar impact on the functional capacity of individuals with type 2 diabetes, due to low cardiorespiratory fitness[19], low physical activity levels [20], and high body mass index (BMI). Individuals with diabetes covered less distance during a 6 minute walk test (6MWT) as compared to age and sex matched controls [21]. However, the specific impact of fatigue on QoL and functional status has not yet been investigated in people with diabetes.

Although there is little research on the specific causes of fatigue in people with diabetes, investigators have developed models that illustrate multiple factors that contribute to fatigue in people with chronic illness [22–26]. These possible contributing factors includes physiological components that mainly cover the underlying disease and the complications which comes with the treatment and the disease and psychological components which includes depression and anxiety [27]. There may also be physiological and psychological contributions to fatigue in people with diabetes. Episodes of hyperglycemia, which is often observed during diabetes treatment, has been suggested by various authors as a particular cause of fatigue [28, 29]. Numerous complications and the comorbidities associated with these complications such as sleep problems [30] or chronic pain [31] can also contribute to fatigue. Most people with type 2 diabetes are overweight [32], and obesity in general may cause higher fatigue levels [33]. Several psychological factors like depression[34] or stress resulting from the diagnosis or management of diabetes may also contribute to fatigue [35–37]. The relationship between fatigue and the possible physiological and psychological factors in individuals with type 2 diabetes is still unknown.

The purpose of this study was to investigate relationship of fatigue with QoL and functional status and to investigate the contributing factors of fatigue in people with type 2 diabetes. Considering the impact of fatigue on QoL and functional status, the findings of the study will help the health care professions to include assessment of fatigue in patients with type 2 diabetes. The findings of contributing factors of fatigue will help clinicians treat and manage fatigue in patients with diabetes.

## Methods

### Design

A cross sectional, correlational study design was used.

The study setting was an academic medical center in the Midwestern United States. Potential study participants were identified either by self-referral in response to study advertisements directed to individuals 40–70 years and diagnosed with type 2 diabetes and distributed around the community, or through a research participant registry program maintained by the University of Kansas Medical Center. Exclusion criteria were 1) known history of stroke, cancer or other central nervous system pathology which may cause additional burden of fatigue besides diabetes, 2) inability to ambulate, or 3) amputation. The study was approved by University of Kansas Medical Centre’s Human Subjects Committee, and participants who were eligible for the study signed an institutionally-approved informed consent form.

#### Fatigue assessment

Fatigue was assessed with the Multidimensional Fatigue Inventory (MFI-20), which consists of five dimensions: general fatigue, physical fatigue, mental fatigue, reduced motivation and reduced activity [38]. The current version of the MFI-20 contains 20 statements which cover different aspects of fatigue. Each of the 5 scales contains 4 items, with scores that range from 1 for "no, that is not true" to 5 for "yes, that is true". For each scale a total score (range 4–20) is calculated by summation of the scores of the individual items, and higher scores mean higher fatigue. The MFI-20 has good reliability and validity [39].

#### Quality of life

Quality of life was assessed using the Audit of Diabetes Dependent Quality of Life (ADDQoL) measure. The ADDQoL is a widely used health status questionnaire comprised of 19 items [40]. The ADDQoL aims to measure individual’s feelings about the impact of diabetes on several life domains, such as work, family, food, and sex. It also determines whether the impact of diabetes on different life domains is positive or negative and the perceived importance of each domain on the person’s QoL. Participants provide both impact (range -3 to +3) and importance (range 0–3) scores. These two scores were multiplied to get a weighted impact score for each applicable domain (range -9 to +9). An average weighted impact (AWI) score is calculated by adding the weighted impact scores for each domain and dividing by the number of applicable domains. Lower scores indicate a greater negative impact of diabetes on QoL. The ADDQoL has been shown to have good reliability and strong validity [40–44].

### Functional Status

Functional status was evaluated by the 6 minute walk test (6MWT), an established measure with high reliability and validity scores[45] and also a strong predictor of morbidity and mortality [46]. In this study, the test was conducted along a straight corridor (100ft) and standardized instructions were followed [47].

#### Potential contributing factors

1. Hyperglycemia was evaluated via glycosylated hemoglobin (A1C) with a finger stick blood test using a disposable A1C analyzer (A1CNow+, Bayer Medical Care; Tarrytown, New York). This test gives an indication of the level of glycemic control over a 3-month period. The accuracy of this test is 99% as reported by the manufacturer.

2. Sleep quality was assessed by using Pittsburg Sleep Quality Index (PSQI). PSQI is a self-report questionnaire assessing sleep quality over a 1 month interval. PSQI generates a global score ranging from 0 to 21. Higher global score indicates worse sleep quality. PSQI has been demonstrated to have good reliability and validity[48] and has been used in individuals with diabetes [49].

3. Pain was assessed by using unmarked 10cm Visual analog scale to indicate the pain level at the moment of testing.

4. Diabetes duration was evaluated by calculating the number of years since diabetes was diagnosed.

5. BMI was calculated by dividing measured weight in kilograms by height in meters^2^ (kg/m^2^).

6. Number of complications (NOC) was calculated by counting the number of “Yes” answers given by participants to a list of complications. List of complications include: heart problems, kidney problems, eye problems, tingling in toes/feet, decreased sexual interest, oral infection, depression, gastroparesis, and sleep problems.

7. Depression was evaluated by Beck’s Depression Inventory-2 (BDI-2). The BDI-2 is a 21-item survey, with each item consisting of a list of four statements arranged in increasing severity about a particular symptom of depression. Scores range 0 from 13 (normal), 14 to 19 (mild depression), 20 to 28 (moderate depression), and 29 to 63 (severe depression).

### Data Analysis

Descriptive statistics (mean, standard deviation, median, and range) of each of the contributing factors (MFI-20, HbA1c, PSQI, VAS, years since diagnosis, BMI, NOC), ADDQoL, and 6MWT were calculated. Assumptions of normality and equal variance were tested by using Kolmogorov-Smirnov Z and Leven’s statistic. Data from two participants was excluded from main statistical analysis because of two outliers in BMI, skewing the data; assumptions were met after excluding the two outliers.

SPSS version 20 was used to perform Pearson product-moment correlation coefficient to determine the relationship of fatigue (general fatigue–MFI-20) with potential contributing factors, ADDQoL and 6MWT.To explore the potential contributing factors, and significant variables in bivariate correlations were included in the multivariable linear regression model. Assumptions of normality, linear relationship, equal variance and multi-collinearity were tested. Decisions on assumptions were made on the residuals of the main outcome variable (MFI-20). Residuals are the differences between the observed value and estimated function value. No transformations were done since the residuals of all outcome variables were following the assumptions of normal distribution. It is more important for the residuals to follow normal distribution as compared to the independent variables (predictors) [50].

Once the regression model was built, an assessment of multicollinearity using variance inflation factors (VIF) was conducted to determine whether or not this issue has resulted in a biased model [51]. Forward stepwise entry was used to enter the 4 variables that were ultimately selected as possible explanatory variables into the regression model.

## Results

Data from 48 participants is presented. Participant characteristics are provided in Table 1 along with mean and standard deviation (SD) of MFI-20 domains. Table 2 presents co-relation analysis of fatigue with QoL/functional measures and potential contributing factors. No significant gender differences were observed in fatigue scores (*P* = 0.25).

**Table 1:** Demographics, mean and SD of MFI-20 domains.

Demographics	Mean (SD); N = 48
Age	59.66 (7.24)
Sex	22 females, 26 males
General Fatigue (MFI-20)	13.29 (3.71)
Physical Fatigue (MFI-20)	12.47 (3.48)
Reduced Activity (MFI-20)	11.50 (3.87)
Reduced Motivation (MFI-20)	10.20 (3.47)
Mental Fatigue (MFI-20)	8.89 (3.89)

**Table 2:** Co-relation analysis of fatigue with functional outcomes and contributing factors.

Pearson correlation (r)						
	Mean (SD),n = 48	General fatigue score (MFI-20)	Physical fatigue score (MFI-20)	Reduced activity score (MFI-20)	Reduced motivation scores (MFI-20)	Mental fatigue scores (MFI-20)
ADDQoL	-2.19 (1.65)	-.445**	-.489**	-0.168	-0.165	-0.269
Functional Status (6MWT) meters	361.06 (73.43)	-.386**	-.535**	-0.199	0.025	-0.176
HbA1C	7.38 (1.85)	-0.218	-0.11	-0.054	-0.187	-0.322
BMI	35.28 (7.35)	.350*	.316*	0.126	0.126	-0.051
Sleep quality (PSQI)	8.12(3.64)	.613**	.512**	.442**	.368*	.351*
Pain (VAS)	2.64(2.37)	.437**	.437**	0.142	0.097	0.205
NOC	3.91(2.11)	0.199	.402**	0.114	0.114	0.201
Years since diagnosis	10.45(7.38)	-0.125	0.002	-0.033	-0.194	-0.155
Depression (BDI-2)	11.54(8.75)	.422**	.460**	.425**	.400**	.512**

*significance at p≤.05

** significance at p≤ .01.

Abbreviations: MFI-20: Multidimensional Fatigue Inventory; ADDQoL: Audit of Diabetes Dependent Quality of Life, 6MWT: 6 Minute Walk Test; HbA1c: glycosylated hemoglobin, BMI: Body Mass Index, PSQI: Pittsburg Sleep Quality Index; VAS: Visual Analog Scale; NOC: Number of complications, BDI-2: Beck’s Depression Inventory

As presented in Table 2, there was a significant inverse relationship between general fatigue (MFI-20) and weighted impact ADDQoL scores, and between general fatigue and 6MWT. When MFI-20 domains were further explored, significant relationships were found between physical fatigue, ADDQoL scores and 6MWT (r = -.489), (r = -.535). Physical fatigue was further significantly related to BMI (r = .316), sleep quality (PSQI) (r = .512), pain (VAS) and NOC (r = .402). Other domains of MFI-20; reduced activity was significantly related to sleep quality (PSQI) (r = .442), depression (BDI-2) (r = .425) reduced motivation was related to sleep quality (PSQI) and depression (BDI-2) (r = .368, r = .400) and mental fatigue was related to HbA1c, sleep quality (PSQI) and depression (BDI-2) (r = -.322, r = .351, r = .512).

There was no significant relationship between NOC and general fatigue. The frequency of each complication on the NOC survey is presented in Table 3. A majority of participants indicated that diabetes affected their toes / feet and nerves in legs / arms. Approximately 40% of participants reported sleep problems, decrease sexual interest, and depression.

**Table 3:** Frequency distribution of complications from NOC.

Question from NOC	Number (%) reporting “yes”
Effect on toes or feet	34 (70.8%)
Effect on nerves in legs or arms	28 (58.3%)
Sleep problems	21 (43.8%)
Decreased sexual interest	21 (43.8%)
Depression	19 (39.6%)
Gastroparesis/delayed gastric emptying	17 (35.4%)
Effect on eyes	16 (33.3%)
Kidney problems	12 (25%)
Heart attack	7 (14.6%)
Wounds on soles of feet	7 (14.6%)
Oral infection	4 (8.3%)

The contributing factors that were found to have significant correlations with general fatigue, BMI, PSQI, VAS and BDI-2, were used as explanatory variables to build the multivariable linear regression model. Forward step wise multiple regression analysis was performed using MFI-20 general fatigue as the dependent variable. Sleep quality, BMI and VAS remained the significant predictors of fatigue in the sample, accounting for 57.3% of variance in fatigue scores. There was no issue of multicollinearity as VIF was close to 1 for all the outcome variables. Depression was not a significant predictor in this model. Table 4 summarizes the results of the regression model.

**Table 4:** Results of forward stepwise multiple regression analysis using General fatigue as dependent variable.

Predictors	Unstandardized Coefficients; B	Standardized Coefficients; β	P value of t-test	R^2^	Correlations		VIF
					Partial	Part	
PSQI	0.563	0.552	0	0.375	0.632	0.534	1.07
PSQI, BMI	0.17	0.337	0.001	0.507	0.456	0.335	1.011
PSQI,BMI,VAS	0.416	0.266	0.013	0.573	0.365	0.256	1.079
BDI-2	-0.017	-0.039	0.753		-0.048	-0.032	1.567

Estimated standardized regression coefficients (β) and variance explained (R^2^) are presented.

## Discussion and Conclusion

The purpose of this study was to investigate the relationship of fatigue with QoL, functional status and to investigate predictors of fatigue in people with type 2 diabetes. Our primary findings revealed the negative relationship of fatigue with QoL and functional status, and revealed that sleep quality, BMI, and pain were the strongest explanatory factors for fatigue. Fatigue in this population is similar in magnitude to chronically ill populations [52]. General fatigue among individuals with type 2 diabetes in this study as measured with MFI-20 was similar to previously reported fatigue scores in women with type 2 diabetes (13.29 ±3.71vs. 12.4 ±7.9).[39]

The results of the present study depicting the negative relationship of fatigue with QoL and functional status are in agreement with previous studies [53]^,^[54]^,^[55]. A negative relationship between fatigue and QoL was previously reported in patients with cancer receiving chemotherapy n = 263 (r = -.63; P < .001) [56]. Goldman et al. reported a negative association of fatigue with 6MWT (with a spearman rank relationship of ρ = .59; P = 0.001) in people with severe multiple sclerosis [53]. In the present study, we found similar significant relationships that have not previously been identified in individuals with diabetes. Diabetes requires extensive management and care, which may be complicated by the acute physical distress of glycemic variability and the distress of diabetes-related complications. The negative relationship found in this study between fatigue, QoL, and functional status is not necessarily a causal relationship. There may be a bi-directional relationship between factors, or the relationship may in fact be caused by an additional factor.

Findings from the present study support the previous studies exploring the relationship of fatigue with sleep quality, pain and BMI in people with diabetes [31]^,^ [57]^,^ [58] and with cancer [59–62]. Sleep is an essential requirement for healthy functioning of human mind and body. Patients with type 2 diabetes suffer with poor sleep quality adversely affecting the glucose control and negatively impacting diabetes self-management in people with diabetes [63],[64]. Poor sleep quality has been identified as a contributing factor to fatigue by the present study. The NOC survey results indicated that sleep was a problem for a large number (43%) of our participants. Poor sleep quality can be due to numerous reasons including; nocturia, polyuria, neuropathy pain, obstructive sleep apnea, hypertension [65]. There is still an uncertainty as to what causes poor sleep quality. More vigorous methods to test sleep quality including testing sleep quality in a sleep lab or sleep clinic might explain the causes of poor sleep quality. Pain was also identified as a contributing factor to fatigue. Pain may result in decreased physical activity performed by those with diabetes [66]. Over 70% of our participants reported nerve problems on NOC survey which may have included pain. Pain can also result in difficulty falling asleep, sleep disturbances caused by pain leading to anxiety and depression, thus additionally deteriorating sleeping disorders, so many patients enter a vicious cycle of sleep deprivation. Sleep deprivation in return results in fatigue, strongly influencing patient’s ability to function and dependence on others in everyday functioning [67]. High BMI has been directly linked to the occurrence of type 2 diabetes and has also been positively associated with fatigue scores of MFI-20 in type 2 diabetic patients [68]. The present study further supports this by identifying high BMI as a contributing factor to fatigue. Obesity is a major risk factor for developing type 2 diabetes. Since, physical activity is one of the corner stone for self-management of diabetes, obesity also impacts the physical activity leading to decrease motility and dependence. This decrease level of function can lead to fatigue, thus additionally affecting the sleep quality.

We did not find a relationship between fatigue and glucose control (as measured by HbA1c), or other indications of diabetic severity (number of complications and years since diagnosis). These findings are consistent with other studies in which only weak [69],[70] or no[71] relationships were found. HbA1c tests the average blood glucose for the last 3 month period. Since the test gives an average of blood glucose, the fluctuations between high and low blood glucose are not captured by the HbA1c test. Fatigue may result from these blood glucose fluctuations or from high/low blood glucose events [72, 73]. Therefore, other measures of blood glucose like fasting blood glucose or continuous glucose monitoring [74] are needed to further explore the relationship of fatigue with blood glucose fluctuations. Considering diabetes severity, no significant relationship of fatigue was found with number of complications and years since diagnosis. Participants had to answer “yes” to a list of questions related to diabetes complications which reduces the chance to catch impact of the complications, for example, heart problems can vary from high blood pressure to angina. Other ways of evaluating complications from diabetes should be included; this includes using diabetes symptom check list or other methods like analyzing side effects of different medications use, screening for nephropathy, retinopathy neuropathy should be performed for future studies. Diabetes duration or years since diagnosis was also not related to fatigue scores. There was a limitation to exploring this variable because of the uncertainty of exact diagnosis year. Diabetes is often diagnosed relatively late, at a point where may patients have already developed complications. Other explanation of lack of relationship of diabetes duration with fatigue scores could be acceptance of the disease, following the management strategies and having the condition for many years and yet not have experienced any complications. Therefore depending on exact year of diagnosis is not a reliable tool.

Depression was not a significant predictor of fatigue in the present study, although it showed a significant correlation with fatigue, and 39% of our participants reported depression on the NOC survey. Fatigue is one of the most prevalent symptom of depression and significant relationship between fatigue and depression has been reported in the past [75],[76]. As the tool used to capture depression in the present study (BDI-2) is a screening tool and may not be sensitive to detecting relationships, this might explain why depression was not a significant predictor of fatigue. More specific depression diagnosis assessment tools, like the patient health questionnaire (PHQ-9), may be required to further test the relationship of depression and fatigue in people with diabetes. Additionally, future investigation should be done regarding depression and treatment of depression, since this can directly affect fatigue levels.

The results of the regression analysis are based on an exploratory regression model based on the sample size limiting number of inclusion factors. A larger sample size would be important for future research to conduct confirmatory regression analysis.

It should be noted that fatigue is an inherently difficult concept to study. The participants could be biased towards their fatigue levels and could be saying what they think the researcher wants to hear because of the direct questioning by the researchers. We tried to minimize this matter by following a strict protocol while asking specific fatigue related questions so that the participants do not over represent presence of fatigue.

The other limitation of the study was small sample size and lack of comparison group. The use of large sample and comparison group using sleep quality/pain/BMI matched people without diabetes could have helped us in identifying the diabetes measures which can influence fatigue in people with diabetes. Future studies should include a control group to help provide a better understanding of diabetes measures influencing fatigue in this population.

Individuals with diabetes often take multiple medications and polypharmacy can also result in a number of complications and side effects, including fatigue. Metformin, which is the most widely used insulin sensitizer, can result in side effects including GI disturbances and elevated homocysteine levels (an amino acid),[77]which may result in chronic fatigue [78]. Additionally, medication use was not investigated in the present study. Insulin treatment can lead to hyper/hypoglycemia. Therefore there is a need to investigate the relationship between the use of medications and fatigue levels. Low levels of physical activity have been strongly associated with fatigue in people with type 2 diabetes [79]. Common barriers to physical activity include obesity [80] and fatigue complaints [81]. Therefore future studies should be done addressing the medications, immobility and lack of exercise.

The results of the present study, depicting high fatigue scores and negative impact of fatigue on QoL and function, support the need for inclusion of symptom assessment in diabetes education. The patients may not bring the fatigue issues during their visits to health care providers, with acute issues taking priority during their visit. It is important for health care providers treating the patient with type 2 diabetes to address their fatigue issues. Health care providers should use complaints of fatigue as a starting point for further evaluation of comorbid conditions. In conclusion, results from the present study suggest that interventions strategies should be developed to reduce the burden of fatigue in individuals with type 2 diabetes. Intervention should focus on addressing healthy sleep habits, weight management and pain management. As this may be an important area for diabetes management, further research is warranted to examine fatigue.

## Supporting Information

S1 File Data. (PDF)
