# Supplementary material for: Fatigue in Type 2 Diabetes: Impact on Quality of Life and Predictors
Source: PLoS One. 2016 Nov 8;11(11):e0165652. doi: 10.1371/journal.pone.0165652 (PMC5100935; doi:10.1371/journal.pone.0165652)
Supplement: S1 File — (PDF) [file pone.0165652.s001.pdf]

Age	Gender	Weight (Kg)	Height (mtrs	mtr²	BMI	Years since	Hba1c	Gen Fat
65	F	87.08	1.52	2.3104	37.69044	5	6.6	13
61	F	62.1	1.64	2.6896	23.08894	25	5.1	5
70	M	100.7	1.72	2.9584	34.03867	10	7.1	12
67	M	105.4	1.69	2.8561	36.90347	6	6.1	9
52	F	139.1	1.74	3.0276	45.94398	15	7.8	18
63	M	121.8	1.86	3.4596	35.20638	8	6.6	13
51	M	88.7	1.74	3.0276	29.29713	1	6.1	14
67	M	101.4	1.78	3.1684	32.00353	15	7.1	10
65	F	96.3	1.63	2.6569	36.24525	3	6	13
51	F	85.27	1.67	2.7889	30.57478	3	8.4	11
54	F	110	1.56	2.4336	45.20053	3	6	16
60	F	132	1.66	2.7556	47.90245	3	5.8	19
70	F	76.2	1.59	2.5281	30.14121	10	6.4	11
47	F	64.3	1.64	2.6896	23.9069	7	7.4	16
59	F	92.1	1.62	2.6244	35.09374	5	5.9	12
64	M	127.1	1.86	3.4596	36.73835	17	9.5	11
54	M	90.4	1.7	2.89	31.28028	12	5.6	14
65	F	114.4	1.6	2.56	44.6875	7	6.2	20
58	M	106.7	1.9	3.61	29.55679	10	9.4	7
60	M	100.3	1.75	3.0625	32.75102	4	7	6
66	M	147.4	1.76	3.0976	47.58523	12	6.2	15
64	M	165.2	1.89	3.5721	46.24731	11	9.2	16
59	M	103.9	1.75	3.0625	33.92653	10	7.6	13
69	F	66.2	1.68	2.8224	23.45522	20	8.5	10
57	M	154	1.83	3.3489	45.98525	6	9.8	13
65	M	98.5	1.75	3.0625	32.16327	6	6.2	14
61	F	63.4	1.65	2.7225	23.28742	1	6.3	11
60	F	98.4	1.62	2.6244	37.49428	26	6.1	15
49	F	105.4	1.69	2.8561	36.90347	7	10	13
66	M	98.8	1.87	3.4969	28.2536	16	7.6	16
61	M	92.1	1.69	2.8561	32.24677	25	8.7	12
47	M	75.8	1.79	3.2041	23.65719	17	5.8	10
48	F	90.9	1.56	2.4336	37.35207	2	7	18
48	F	124.7	1.66	2.7556	45.2533	17	12.6	11
64	M	94	1.83	3.3489	28.06892	6	5.5	13
69	M	130	1.82	3.3124	39.24647	27		14
62	M	80.5	1.64	2.6896	29.9301	12	10.8	19
51	F	137.8	1.63	2.6569	51.86496	4	10.8	10
65	M	107.2	1.81	3.2761	32.72183	8	6.2	10
65	M	160.5	1.9	3.61	44.45983	1	6.2	17
65	M	105.9	1.76	3.0976	34.18776	20	9.1	13
60	F	81.8	1.61	2.5921	31.55742	11	11.6	6
65	F	99.8	1.71	2.9241	34.13016	5	4.8	19
67	M	122	1.73	2.9929	40.76314	4	5.6	19
58	F	91.8	1.67	2.7889	32.9162	19	8.3	19
45	M	102	1.87	3.4969	29.16869	1	9.1	12

44	M	95	1.8	3.24	29.32099	19	5.8	18
61	F	100.3	1.52	2.3104	43.4124	20	5.7	12

Phy Fat	Red Act	Red Mot	Men Fat	Tot MFI	VAS	PSQI	ADDQoL	BDI-2
16	6	10	14	59	1.5	10	-3.47	9
10	7	6	10	38	0	6	-1.117	23
13	12	10	12	59	2	10	-2	12
9	11	9	13	51	2	1	-2.578	8
15	11	16	11	71	4.5	8	-2.764	26
10	8	8	9	48	2.7	8	-1.5	6
12	11	12	7	56	5	7	-3.05	14
11	11	8	7	47	3	5	-1.833	3
11	12	13	12	61	4.5	14	-1.63	29
12	14	13	5	55	0	10	-1.33	2
15	20	15	14	80	3.5	9	-1.63	35
14	8	12	4	57	5	9	-3.444	21
8	9	6	6	40	7	8	-0.833	7
5	5	14	6	46	0	11	-0.42	5
11	9	8	5	45	2	5	-1.16	5
10	7	9	5	42	0	6	-1.16	6
14	13	10	9	60	7	13	-3.84	18
19	7	8	4	58	7.5	6	-3.68	15
8	12	10	5	42	1	2	-3.44	2
5	4	5	6	26	1.5	2	-0.5	2
14	16	16	12	73	1	7	-5.1	9
13	14	11	4	58	2.7	10	-3	7
12	12	9	6	52	0	12	-0.79	7
13	13	8	6	50	4	6	-0.947	2
15	10	12	6	56	6.2	5	-2.84	7
10	16	16	15	71	3.3	5	-0.67	16
11	11	12	13	58	6.5	8	-3.83	7
14	15	10	9	63	1.2	8	-2.28	11
13	17	16	8	67	0	13	-2.32	20
14	15	15	10	70	1	16	-4.833	7
10	5	4	5	36	1.4	5	-5.28	7
6	8	8	8	40	1.3	5	-1.26	4
19	14	9	18	78	8	17	-8.06	39
9	6	7	4	37	1.3	5	-0.84	1
13	9	10	9	54	1.4	7	-0.94	15
17	15	12	8	66	3.4	7	-1.5	17
18	18	12	15	82	0.8	12	-2.22	10
9	11	5	5	40	0	7	-0.21	6
9	16	15	10	60	0	7	-0.63	21
16	15	10	11	69	3	12	-2.33	14
13	13	11	11	61	3.5	5	-0.84	5
9	7	4	7	33	0.3	5	-0.58	7
16	14	15	19	83	2.8	12	-3.56	12
16	15	11	10	71	2.5	9	-1.39	15
18	19	13	15	84	7.36	12	-2.33	22
15	10	4	6	47	0	5	0	7

17	12	5	4	56	4.5	14	-5.17	10
12	9	8	9	50	0	4	-0.105	1

NOC	6 Min
5	259.2
5	495.6
4	319
7	365
4	276
2	442
6	498.2
6	405
3	304.1
7	435.5
3	422
4	394.8
2	308
5	400.1
1	413.1
4	464.5
5	302.9
3	262.3
1	428.5
1	469.8
6	345.4
4	376.8
2	418.2
5	193.1
7	332.8
0	463.5
3	388.4
1	333.9
5	403.2
6	391
5	401.3
0	423.8
8	341.6
2	376.2
5	420.5
5	314.8
5	284.9
0	393
3	347.6
3	347.4
2	427.4
7	269.6
7	287.3
4	361.4
4	236
4	369

6	185.1
1	331.9
